# 1-(4,6-Dimethyl­pyrimidin-2-yl)-3-(3,5-dinitro­benzo­yl)thio­urea monohydrate

**DOI:** 10.1107/S1600536811012323

**Published:** 2011-04-13

**Authors:** Sohail Saeed, Naghmana Rashid, Wing-Tak Wong, Rizwan Hussain

**Affiliations:** a Department of Chemistry, Research Complex, Allama Iqbal Open University, Islamabad 44000, Pakistan; b Department of Chemistry, The University of Hong Kong, Pokfulam Road, Pokfulam, Hong Kong SAR, People’s Republic of China; c National Engineering & Scientific Commission, PO Box 2801, Islamabad, Pakistan

## Abstract

The organic molecule in the title mol­ecule, C_14_H_12_N_6_O_5_S·H_2_O, is roughly planar with a maximum deviation of 0.156 (2) Å. An intra­molecular N—H⋯N hydrogen bond occurs. In the crystal, inter­molecular N—H⋯O and O—H⋯O hydrogen-bonding inter­actions connect the mol­ecules into a two-dimensional network that lies parallel to (101).

## Related literature

For background to this study and our previous work on the structural chemistry of *N*,*N*′-disubstituted thio­urea, see: Saeed *et al.* (2011 ▶).
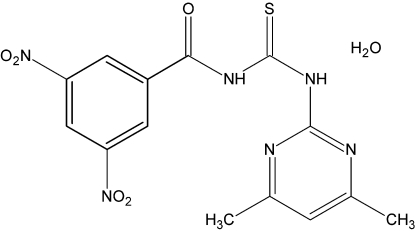


## Experimental

### 

#### Crystal data

C_14_H_12_N_6_O_5_S·H_2_O
*M* *_r_* = 394.37
Monoclinic, 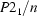

*a* = 6.7892 (6) Å
*b* = 10.1823 (9) Å
*c* = 24.267 (2) Å
β = 92.901 (1)°
*V* = 1675.4 (3) Å^3^
*Z* = 4
Mo *K*α radiation
μ = 0.24 mm^−1^
*T* = 297 K
0.40 × 0.16 × 0.14 mm

#### Data collection

Bruker SMART 1000 CCD diffractometer
Absorption correction: multi-scan (*SADABS*; Sheldrick, 2004 ▶) *T*_min_ = 0.909, *T*_max_ = 0.967
9067 measured reflections
2942 independent reflections
2499 reflections with *I* > 2σ(*I*)
*R*_int_ = 0.020

#### Refinement

*R*[*F* ^2^ > 2σ(*F* ^2^)] = 0.038
*wR*(*F* ^2^) = 0.105
*S* = 1.06
2942 reflections
263 parameters
H atoms treated by a mixture of independent and constrained refinement
Δρ_max_ = 0.20 e Å^−3^
Δρ_min_ = −0.23 e Å^−3^

### 

Data collection: *SMART* (Bruker, 2007 ▶); cell refinement: *SAINT* (Bruker, 2007 ▶); data reduction: *SAINT*; program(s) used to solve structure: *SIR92* (Altomare *et al.*, 1994 ▶); program(s) used to refine structure: *SHELXL97* (Sheldrick, 2008 ▶); molecular graphics: *Mercury* (Macrae *et al.*, 2008 ▶); software used to prepare material for publication: *SHELXL97*.

## Supplementary Material

Crystal structure: contains datablocks global, I. DOI: 10.1107/S1600536811012323/pv2402sup1.cif

Structure factors: contains datablocks I. DOI: 10.1107/S1600536811012323/pv2402Isup2.hkl

Additional supplementary materials:  crystallographic information; 3D view; checkCIF report

## Figures and Tables

**Table 1:** Hydrogen-bond geometry (Å, °)

*D*—H⋯*A*	*D*—H	H⋯*A*	*D*⋯*A*	*D*—H⋯*A*
N4—H4*N*⋯O6	0.88 (2)	1.97 (2)	2.849 (2)	171.9 (19)
N3—H3*N*⋯N6	0.83 (2)	2.00 (2)	2.705 (2)	141.8 (19)
O6—H6*A*⋯O3^i^	0.82 (4)	2.32 (4)	3.119 (3)	168 (4)
O6—H6*B*⋯O2^ii^	0.73 (4)	2.44 (4)	3.143 (3)	164 (4)
O6—H6*B*⋯O1^ii^	0.73 (4)	2.62 (4)	3.248 (3)	146 (4)

Symmetry codes: (i) 
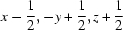
; (ii) 
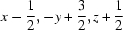
.

**Fig. 1.**
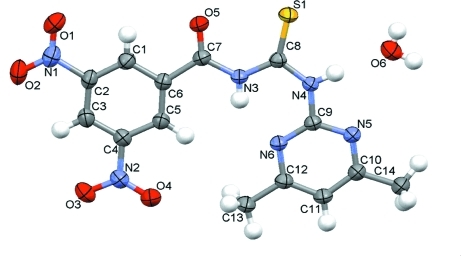
An ORTEP plot of the title molecule drawn with 50% probability thermal ellipsoids showing the atom numbering scheme.

**Fig. 2.**
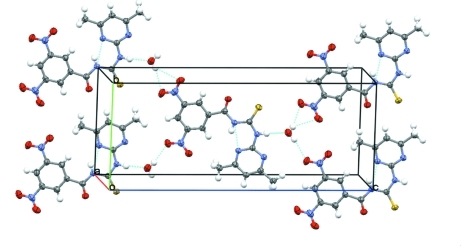
Packing diagram of the title compound viewed down the a axis.

## supplementary crystallographic information

### Comment

The background to this study has been described in our previous work on the structural chemistry of N,N'-disubstituted thiourea (Saeed *et al.*, 2011). In continuation of our studies, the crystal structure of the title compound, is presented in this paper.

In the title molecule (Fig. 1), the nitro groups N1/O1/O2 and N2/O3/O4 are oriented are 5.05 (11) and 9.40 (15)°, respectively, with rest to the mean-plane of the phenyl ring C1—C6. Moreover, the 4,6-dimethyl-pyrimidinyl ring plane, C9—C14/N5/N6, makes a dihedral angle of 1.31 (5)° with the the plane formed by the atoms C7/O5/N3/C8/S1/N4.

The structure is stabilized by intra-molecular N—H···O and N—H···N hydrogen bonding interactions. The inter-molecular hydrogen bonds O—H···O link the molecules into a two dimensinal network (Tab. 1 and Fig. 2).

### Experimental

A solution of 3,5-dinitrobenzoyl chloride (0.01 mol) in anhydrous acetone (75 ml) and 3% tetrabutylammonium bromide as a phase-transfer catalyst in anhydrous acetone was added dropwise to a suspension of dry potassium thiocyanate (0.01 mol) in acetone (50 ml) and the reaction mixture was refluxed for 50 min. After cooling to room temperature, a solution of 2-amino-4,6-dimethylpyrimidine (0.01 mol) in anhydrous acetone (25 ml) was added dropwise and the resulting mixture refluxed for 3 h. Hydrochloric acid (0.1 N, 300 ml) was added, and the solution was filtered. The solid product was washed with water and purified by re-crystallization from ethanol.

### Refinement

All of the C-bound H atoms were observable from difference Fourier map but were placed at geometrically idealized positions with C—H = 0.93 and 0.96Å for phenyl and methyl H-atoms, respectively. The C-bound H-atoms were refined using riding model with *U*_iso_(H) = 1.2*U*_eq_(C). Both the N– and O-bound H-atoms were located from difference Fourier map and refined isotropically.

### Crystal data

**Table:**

C_14_H_12_N_6_O_5_S·H_2_O	*F*(000) = 816
*M**_r_* = 394.37	*D*_x_ = 1.563 Mg m^−^^3^
Monoclinic, *P*2_1_/*n*	Mo *K*α radiation, λ = 0.71073 Å
Hall symbol: -P 2yn	Cell parameters from 11914 reflections
*a* = 6.7892 (6) Å	θ = 2.0–25.0°
*b* = 10.1823 (9) Å	µ = 0.24 mm^−^^1^
*c* = 24.267 (2) Å	*T* = 297 K
β = 92.901 (1)°	Prism, yellow
*V* = 1675.4 (3) Å^3^	0.40 × 0.16 × 0.14 mm
*Z* = 4	

### Data collection

**Table:**

Bruker SMART 1000 CCD diffractometer	2942 independent reflections
Radiation source: fine-focus sealed tube	2499 reflections with *I* > 2σ(*I*)
graphite	*R*_int_ = 0.020
ω scans	θ_max_ = 25.0°, θ_min_ = 2.6°
Absorption correction: multi-scan (*SADABS*; Sheldrick, 2004)	*h* = −8→8
*T*_min_ = 0.909, *T*_max_ = 0.967	*k* = −11→12
9067 measured reflections	*l* = −23→28

### Refinement

**Table:**

Refinement on *F*^2^	Secondary atom site location: difference Fourier map
Least-squares matrix: full	Hydrogen site location: inferred from neighbouring sites
*R*[*F*^2^ > 2σ(*F*^2^)] = 0.038	H atoms treated by a mixture of independent and constrained refinement
*wR*(*F*^2^) = 0.105	*w* = 1/[σ^2^(*F*_o_^2^) + (0.0466*P*)^2^ + 0.9069*P*] where *P* = (*F*_o_^2^ + 2*F*_c_^2^)/3
*S* = 1.06	(Δ/σ)_max_ = 0.003
2942 reflections	Δρ_max_ = 0.20 e Å^−^^3^
263 parameters	Δρ_min_ = −0.23 e Å^−^^3^
0 restraints	Extinction correction: *SHELXL97* (Sheldrick, 2008), Fc^*^=kFc[1+0.001xFc^2^λ^3^/sin(2θ)]^-1/4^
Primary atom site location: structure-invariant direct methods	Extinction coefficient: 0.0044 (6)

### Special details

**Table:**

Geometry. All e.s.d.'s (except the e.s.d. in the dihedral angle between two l.s. planes) are estimated using the full covariance matrix. The cell e.s.d.'s are taken into account individually in the estimation of e.s.d.'s in distances, angles and torsion angles; correlations between e.s.d.'s in cell parameters are only used when they are defined by crystal symmetry. An approximate (isotropic) treatment of cell e.s.d.'s is used for estimating e.s.d.'s involving l.s. planes.
Refinement. Refinement of *F*^2^ against ALL reflections. The weighted *R*-factor *wR* and goodness of fit *S* are based on *F*^2^, conventional *R*-factors *R* are based on *F*, with *F* set to zero for negative *F*^2^. The threshold expression of *F*^2^ > σ(*F*^2^) is used only for calculating *R*-factors(gt) *etc*. and is not relevant to the choice of reflections for refinement. *R*-factors based on *F*^2^ are statistically about twice as large as those based on *F*, and *R*- factors based on ALL data will be even larger.The structure was solved by direct methods (*SHELXS97*) and expanded using Fourier techniques. All non-H atoms were refined anisotropically.All of the C-bound H atoms are observable from difference Fourier map but are all placed at geometrical positions with C—H = 0.93 and 0.96Å for phenyl and methyl H-atoms. All C-bound H-atoms are refined using riding model with *U*_iso_(H) = 1.2*U*_eq_(Carrier). Both the N– and O-bound H-atoms were located from difference Fourier map and refined isotropically.Highest peak is 0.20 at (0.0792, 0.5848, 0.1447) [0.83Å from Hl4C] Deepest hole is -0.23 at (0.0397, 0.8302, 0.0937) [0.74Å from Sl]

### Fractional atomic coordinates and isotropic or equivalent isotropic displacement parameters (Å^2^)

**Table:**

	*x*	*y*	*z*	*U*_iso_*/*U*_eq_	
S1	0.64692 (10)	0.68130 (5)	0.59394 (2)	0.05020 (19)	
O1	0.9131 (3)	0.89155 (18)	0.30790 (8)	0.0777 (6)	
O2	1.0041 (4)	0.7610 (2)	0.24468 (8)	0.0851 (7)	
O3	0.9757 (3)	0.29505 (18)	0.27197 (7)	0.0759 (6)	
O4	0.8719 (4)	0.21594 (17)	0.34703 (7)	0.0802 (6)	
O5	0.7636 (3)	0.73999 (15)	0.48300 (6)	0.0608 (5)	
O6	0.5934 (4)	0.4521 (2)	0.70525 (8)	0.0692 (6)	
H6A	0.561 (5)	0.395 (4)	0.7269 (16)	0.108 (14)*	
H6B	0.569 (6)	0.512 (4)	0.7202 (16)	0.108 (15)*	
N1	0.9439 (3)	0.7823 (2)	0.29029 (8)	0.0563 (5)	
N2	0.9151 (3)	0.30735 (18)	0.31844 (8)	0.0519 (5)	
N3	0.7393 (2)	0.52460 (16)	0.50834 (6)	0.0338 (4)	
H3N	0.753 (3)	0.447 (2)	0.4988 (8)	0.035 (6)*	
N4	0.6648 (2)	0.42786 (15)	0.59103 (7)	0.0354 (4)	
H4N	0.632 (3)	0.440 (2)	0.6254 (10)	0.037 (5)*	
N5	0.6445 (3)	0.21615 (16)	0.61919 (7)	0.0405 (4)	
N6	0.7339 (2)	0.26275 (15)	0.52709 (6)	0.0346 (4)	
C1	0.8532 (3)	0.6919 (2)	0.37878 (8)	0.0395 (5)	
H1	0.8383	0.7775	0.3913	0.047*	
C2	0.9061 (3)	0.6685 (2)	0.32582 (8)	0.0416 (5)	
C3	0.9278 (3)	0.5444 (2)	0.30505 (8)	0.0437 (5)	
H3	0.9645	0.5305	0.2691	0.052*	
C4	0.8928 (3)	0.4414 (2)	0.33990 (8)	0.0399 (5)	
C5	0.8407 (3)	0.45841 (19)	0.39404 (8)	0.0363 (4)	
H5	0.8188	0.3865	0.4166	0.044*	
C6	0.8221 (3)	0.58592 (19)	0.41368 (8)	0.0341 (4)	
C7	0.7720 (3)	0.62534 (18)	0.47127 (8)	0.0362 (4)	
C8	0.6868 (3)	0.54090 (18)	0.56219 (8)	0.0330 (4)	
C9	0.6830 (3)	0.29543 (18)	0.57718 (8)	0.0331 (4)	
C10	0.6548 (3)	0.0876 (2)	0.60875 (8)	0.0417 (5)	
C11	0.7017 (3)	0.04196 (19)	0.55717 (8)	0.0429 (5)	
H11	0.7055	−0.0477	0.5499	0.051*	
C12	0.7424 (3)	0.13190 (19)	0.51685 (8)	0.0371 (4)	
C13	0.7985 (4)	0.0930 (2)	0.46069 (8)	0.0496 (6)	
H13A	0.9262	0.1281	0.4539	0.060*	
H13B	0.7032	0.1269	0.4338	0.060*	
H13C	0.8021	−0.0010	0.4581	0.060*	
C14	0.6125 (4)	−0.0021 (2)	0.65554 (9)	0.0600 (7)	
H14A	0.6208	0.0462	0.6895	0.072*	
H14B	0.7073	−0.0722	0.6573	0.072*	
H14C	0.4824	−0.0381	0.6498	0.072*	

### Atomic displacement parameters (Å^2^)

**Table:**

	*U*^11^	*U*^22^	*U*^33^	*U*^12^	*U*^13^	*U*^23^
S1	0.0823 (4)	0.0302 (3)	0.0389 (3)	0.0073 (2)	0.0114 (3)	−0.0046 (2)
O1	0.1246 (17)	0.0442 (10)	0.0658 (12)	−0.0034 (10)	0.0201 (11)	0.0142 (9)
O2	0.1375 (18)	0.0706 (13)	0.0507 (11)	−0.0010 (12)	0.0386 (11)	0.0165 (9)
O3	0.1253 (16)	0.0596 (11)	0.0457 (10)	0.0000 (10)	0.0324 (10)	−0.0116 (8)
O4	0.1496 (19)	0.0412 (9)	0.0524 (11)	−0.0108 (11)	0.0316 (11)	−0.0044 (8)
O5	0.1047 (13)	0.0309 (8)	0.0490 (9)	−0.0032 (8)	0.0266 (9)	−0.0014 (7)
O6	0.1083 (16)	0.0545 (11)	0.0466 (10)	0.0065 (11)	0.0221 (10)	−0.0072 (9)
N1	0.0725 (14)	0.0516 (12)	0.0452 (11)	−0.0031 (10)	0.0085 (10)	0.0142 (9)
N2	0.0741 (13)	0.0450 (11)	0.0374 (10)	−0.0029 (9)	0.0101 (9)	−0.0037 (8)
N3	0.0430 (9)	0.0269 (8)	0.0320 (8)	0.0018 (7)	0.0057 (7)	−0.0014 (7)
N4	0.0495 (10)	0.0294 (8)	0.0278 (8)	0.0022 (7)	0.0070 (7)	−0.0019 (6)
N5	0.0561 (10)	0.0331 (9)	0.0327 (9)	−0.0012 (7)	0.0063 (7)	0.0004 (7)
N6	0.0429 (9)	0.0296 (8)	0.0315 (8)	0.0024 (7)	0.0038 (7)	−0.0008 (7)
C1	0.0421 (11)	0.0363 (11)	0.0403 (11)	−0.0004 (8)	0.0036 (9)	0.0033 (8)
C2	0.0458 (11)	0.0443 (12)	0.0349 (11)	−0.0030 (9)	0.0042 (9)	0.0102 (9)
C3	0.0495 (12)	0.0506 (12)	0.0316 (10)	−0.0043 (10)	0.0071 (9)	0.0013 (9)
C4	0.0459 (11)	0.0409 (11)	0.0330 (10)	−0.0012 (9)	0.0036 (8)	−0.0025 (8)
C5	0.0390 (10)	0.0375 (10)	0.0325 (10)	−0.0035 (8)	0.0033 (8)	0.0020 (8)
C6	0.0337 (10)	0.0364 (10)	0.0323 (10)	−0.0009 (8)	0.0025 (7)	0.0020 (8)
C7	0.0409 (11)	0.0305 (10)	0.0374 (10)	−0.0013 (8)	0.0041 (8)	−0.0001 (8)
C8	0.0351 (10)	0.0317 (9)	0.0321 (10)	0.0014 (8)	0.0013 (8)	−0.0011 (8)
C9	0.0369 (10)	0.0304 (9)	0.0320 (10)	0.0008 (8)	0.0023 (8)	0.0002 (8)
C10	0.0557 (12)	0.0342 (10)	0.0353 (10)	−0.0034 (9)	0.0044 (9)	0.0017 (8)
C11	0.0602 (13)	0.0287 (10)	0.0403 (11)	0.0009 (9)	0.0073 (9)	−0.0020 (8)
C12	0.0438 (11)	0.0326 (10)	0.0350 (10)	0.0028 (8)	0.0031 (8)	−0.0024 (8)
C13	0.0744 (15)	0.0351 (11)	0.0403 (12)	0.0044 (10)	0.0132 (11)	−0.0043 (9)
C14	0.105 (2)	0.0363 (12)	0.0400 (12)	−0.0073 (12)	0.0160 (12)	0.0025 (10)

### Geometric parameters (Å, °)

**Table:**

S1—C8	1.6528 (19)	C1—C6	1.395 (3)
O1—N1	1.213 (3)	C1—H1	0.9300
O2—N1	1.218 (3)	C2—C3	1.372 (3)
O3—N2	1.226 (2)	C3—C4	1.375 (3)
O4—N2	1.206 (2)	C3—H3	0.9300
O5—C7	1.204 (2)	C4—C5	1.389 (3)
O6—H6A	0.82 (4)	C5—C6	1.391 (3)
O6—H6B	0.73 (4)	C5—H5	0.9300
N1—C2	1.475 (3)	C6—C7	1.509 (3)
N2—C4	1.472 (3)	C10—C11	1.387 (3)
N3—C8	1.382 (2)	C10—C14	1.496 (3)
N3—C7	1.390 (2)	C11—C12	1.379 (3)
N3—H3N	0.83 (2)	C11—H11	0.9300
N4—C8	1.359 (2)	C12—C13	1.487 (3)
N4—C9	1.397 (2)	C13—H13A	0.9600
N4—H4N	0.88 (2)	C13—H13B	0.9600
N5—C9	1.337 (2)	C13—H13C	0.9600
N5—C10	1.336 (3)	C14—H14A	0.9600
N6—C9	1.323 (2)	C14—H14B	0.9600
N6—C12	1.357 (2)	C14—H14C	0.9600
C1—C2	1.373 (3)		
			
H6A—O6—H6B	101 (4)	C1—C6—C7	113.88 (17)
O1—N1—O2	123.7 (2)	O5—C7—N3	123.49 (18)
O1—N1—C2	118.43 (19)	O5—C7—C6	119.51 (17)
O2—N1—C2	117.9 (2)	N3—C7—C6	117.00 (16)
O4—N2—O3	123.56 (19)	N4—C8—N3	115.18 (16)
O4—N2—C4	118.69 (17)	N4—C8—S1	117.87 (14)
O3—N2—C4	117.75 (18)	N3—C8—S1	126.94 (14)
C8—N3—C7	125.51 (17)	N6—C9—N5	128.26 (17)
C8—N3—H3N	114.5 (14)	N6—C9—N4	119.63 (16)
C7—N3—H3N	119.9 (14)	N5—C9—N4	112.11 (16)
C8—N4—C9	132.86 (16)	N5—C10—C11	121.06 (18)
C8—N4—H4N	114.2 (14)	N5—C10—C14	116.15 (18)
C9—N4—H4N	112.9 (14)	C11—C10—C14	122.79 (19)
C9—N5—C10	115.67 (17)	C12—C11—C10	118.78 (18)
C9—N6—C12	115.51 (16)	C12—C11—H11	120.6
C2—C1—C6	119.31 (19)	C10—C11—H11	120.6
C2—C1—H1	120.3	N6—C12—C11	120.68 (17)
C6—C1—H1	120.3	N6—C12—C13	116.40 (17)
C1—C2—C3	122.81 (18)	C11—C12—C13	122.92 (18)
C1—C2—N1	118.21 (19)	C12—C13—H13A	109.5
C3—C2—N1	118.97 (18)	C12—C13—H13B	109.5
C2—C3—C4	116.83 (18)	H13A—C13—H13B	109.5
C2—C3—H3	121.6	C12—C13—H13C	109.5
C4—C3—H3	121.6	H13A—C13—H13C	109.5
C3—C4—C5	123.19 (19)	H13B—C13—H13C	109.5
C3—C4—N2	117.74 (17)	C10—C14—H14A	109.5
C5—C4—N2	119.06 (17)	C10—C14—H14B	109.5
C4—C5—C6	118.16 (17)	H14A—C14—H14B	109.5
C4—C5—H5	120.9	C10—C14—H14C	109.5
C6—C5—H5	120.9	H14A—C14—H14C	109.5
C5—C6—C1	119.68 (17)	H14B—C14—H14C	109.5
C5—C6—C7	126.44 (17)		

### Hydrogen-bond geometry (Å, °)

**Table:**

*D*—H···*A*	*D*—H	H···*A*	*D*···*A*	*D*—H···*A*
N4—H4N···O6	0.88 (2)	1.97 (2)	2.849 (2)	171.9 (19)
N3—H3N···N6	0.83 (2)	2.00 (2)	2.705 (2)	141.8 (19)
O6—H6A···O3^i^	0.82 (4)	2.32 (4)	3.119 (3)	168 (4)
O6—H6B···O2^ii^	0.73 (4)	2.44 (4)	3.143 (3)	164 (4)
O6—H6B···O1^ii^	0.73 (4)	2.62 (4)	3.248 (3)	146 (4)

Symmetry codes: (i) *x*−1/2, −*y*+1/2, *z*+1/2; (ii) *x*−1/2, −*y*+3/2, *z*+1/2.
